# Minimally Invasive Approach to Subdural Hematoma Treatment Using IRRAflow Catheter and Middle Meningeal Artery Embolization

**DOI:** 10.7759/cureus.13979

**Published:** 2021-03-18

**Authors:** Ryan M Hess, Timothy E OConnor, Asham Khan, Adnan H Siddiqui, Jason Davies

**Affiliations:** 1 Neurosurgery, University at Buffalo, Buffalo, USA; 2 Neurosurgery, Jacobs School of Medicine and Biomedical Sciences, Buffalo, USA

**Keywords:** chronic subdural, mma embolization, irraflow

## Abstract

Chronic subdural hematoma (cSDH) is a common neurosurgical pathology that usually occurs in the seventh decade of life. Patients can present with mental status changes, focal neurologic deficits, seizures, headaches, or may be asymptomatic. Recurrence is common. In order to address this problem with the treatment of cSDH, many studies exist that compare the effectiveness of various treatment modalities. Two recently developed treatment options of cSDH include middle meningeal artery (MMA) embolization and use of self-irrigating catheter systems. To our knowledge there have been no reported cases of combining the use of these new treatments. What follows is a case report of a 72-year-old patient with recurrent cSDH following MMA embolization who underwent minimally invasive surgical drainage of his hematoma using an IRRAflow catheter (IRRAS, San Diego, CA, USA).

## Introduction

Chronic subdural hematoma (cSDH) is a common neurosurgical pathology. Incidence is estimated at around 1.7 per 100,000 people per year, most commonly occurring in the seventh decade of life [1]. Patients can present with mental status changes, focal neurologic deficits, seizures, headaches, or may be asymptomatic [2]. Recurrence is common, estimated as high as 33% in some series [3]. In order to address this problem with the treatment of cSDH, many studies exist that compare the effectiveness of various treatment modalities. Two recently developed treatment options of cSDH include middle meningeal artery embolization and use of self-irrigating catheter systems. 

Middle meningeal artery embolization attempts to address the underlying pathology of cSDH by preventing the formation of fragile capillaries within the dural border cell layer which can rupture and lead to continued hemorrhage [4]. Though the effectiveness of this treatment is still being investigated in randomized clinical trials, preliminary findings from case series and retrospective analysis have been promising. The recurrent rate following middle meningeal artery (MMA) embolization has been reported to be between 2.1% and 3.6% [5]. 

Self-irrigating catheter systems such as the IRRAflow system (IRRAS, San Diego, CA, USA) have recently been trialed in the treatment of cSDH. One case series published by Davies et al. using these catheters in six patients had promising results. They report a decrease in average length of stay from six days to 2.83 days and no complications [6]. 

To our knowledge there have been no reported cases of combining the use of these new treatments. What follows is a case report in which a 72-year-old patient with recurrent cSDH following MMA embolization underwent minimally invasive surgical drainage of his hematoma using an IRRAflow catheter. In addition, we include follow-up data from three more patients who underwent the same treatment to compare our length of stay and recurrence rates to other studies. 

## Case presentation

**Figure 1:**
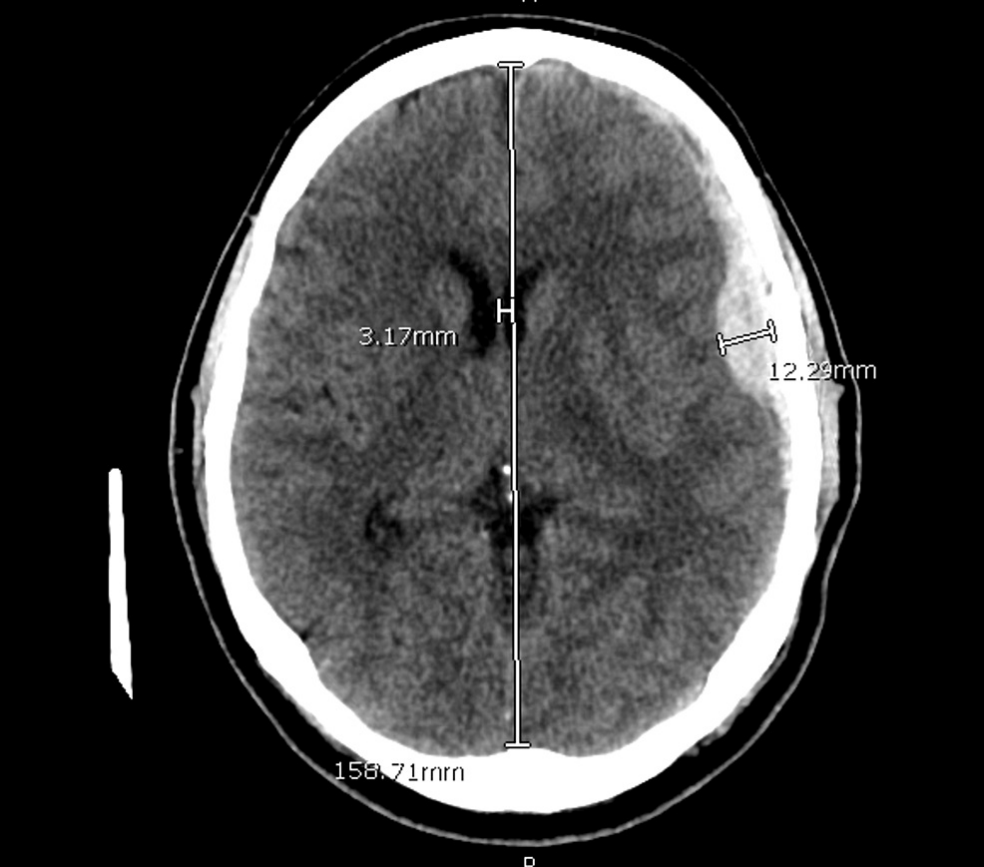
Initial Presentation

The patient was a 72-year-old male with previous medical history of atrial fibrillation on apixaban, hypertension, hyperlipidemia, and prior basal cell carcinoma status post resection who presented with headache. The patient reported earlier on the day of presentation he awoke around 6:00 AM with a diffuse throbbing headache, approximately 5 out of 10 in intensity. He initially tried to manage it conservatively, but he began to vomit and decided to present to an outside emergency room. At the outside facility a dry computed tomography (CT) of his head was performed which demonstrated an acute left subdural hematoma (Figure 1). He was subsequently given prothrombin complex concentrate (PCC) and transferred to our facility. 

On further history the patient endorsed a history of similar headaches in the past that would last for days and were associated with pulsatile tinnitus. He was neurologically intact on initial examination. Given the history of pulsatile tinnitus a diagnostic angiogram was performed to rule out any underlying vascular malformation. This was negative. A magnetic resonance imaging (MRI) of the brain with and without contrast was also performed and was negative for any underlying metastatic lesion. 

**Figure 2:**
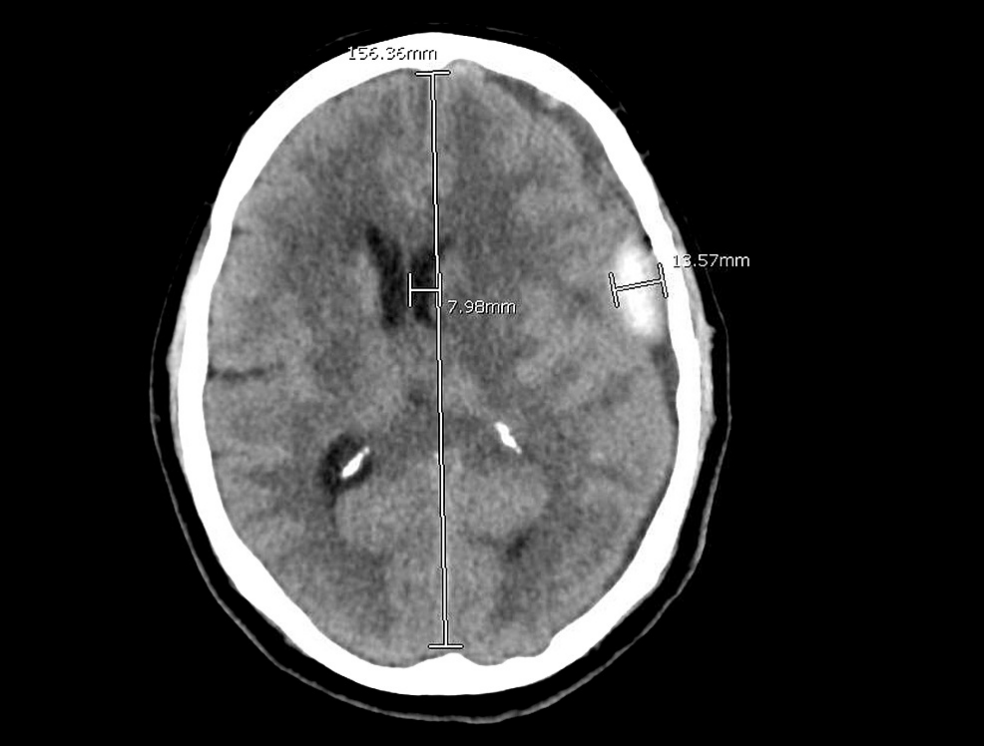
Post-embolization

The patient remained neurologically with improved headaches. The patient was sent home after serial imaging was completed and stable. He was brought back electively for left-sided middle meningeal artery embolization approximately two weeks later. After the completion of this procedure he was discharged home the following day after repeat imaging was obtained and again stable (Figure 2).

**Figure 3:**
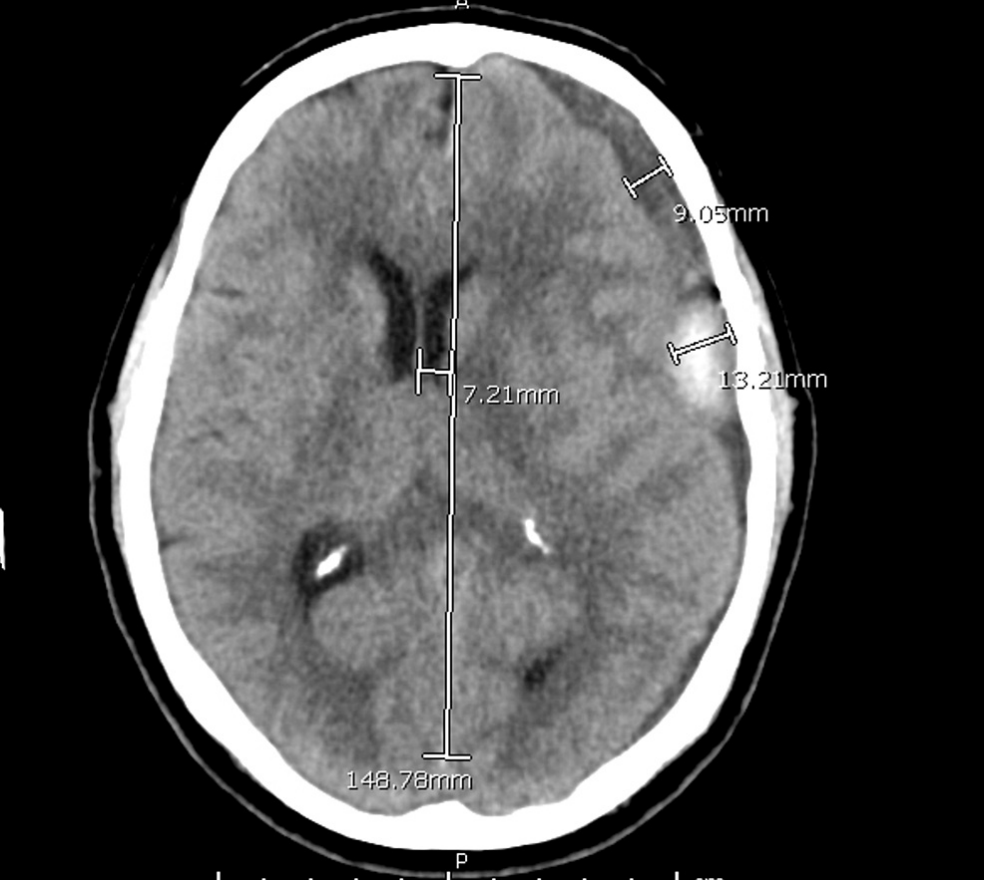
Pre-operative Image

He returned the following evening with a transient episode of speech difficulty that resolved on arrival to the emergency room. Head imaging obtained was unchanged. His levetiracetam dosage was increased to treat seizures and he was sent home with plans to follow up with him in clinic later in the week. The following day he presented again with the same complaints. Head CT at that time again was stable (Figure 3), however, given his persistent symptoms and unresolved subdural hematoma, surgical intervention was performed. 

The patient was transported to the operating room and induced under general anesthesia. He was positioned supine and prepped and draped in sterile fashion. A 4 cm left frontal incision was made and a small craniotomy was performed. The IRRAflow system was primed and tunneled into the incision. The dura was opened and the IRRAflow catheter was then placed in the subdural space. The IRRAflow system was turned on, and subdural hematoma was noted to be draining through the catheter into the collection system. The wound was then closed in standard fashion. 

**Figure 4:**
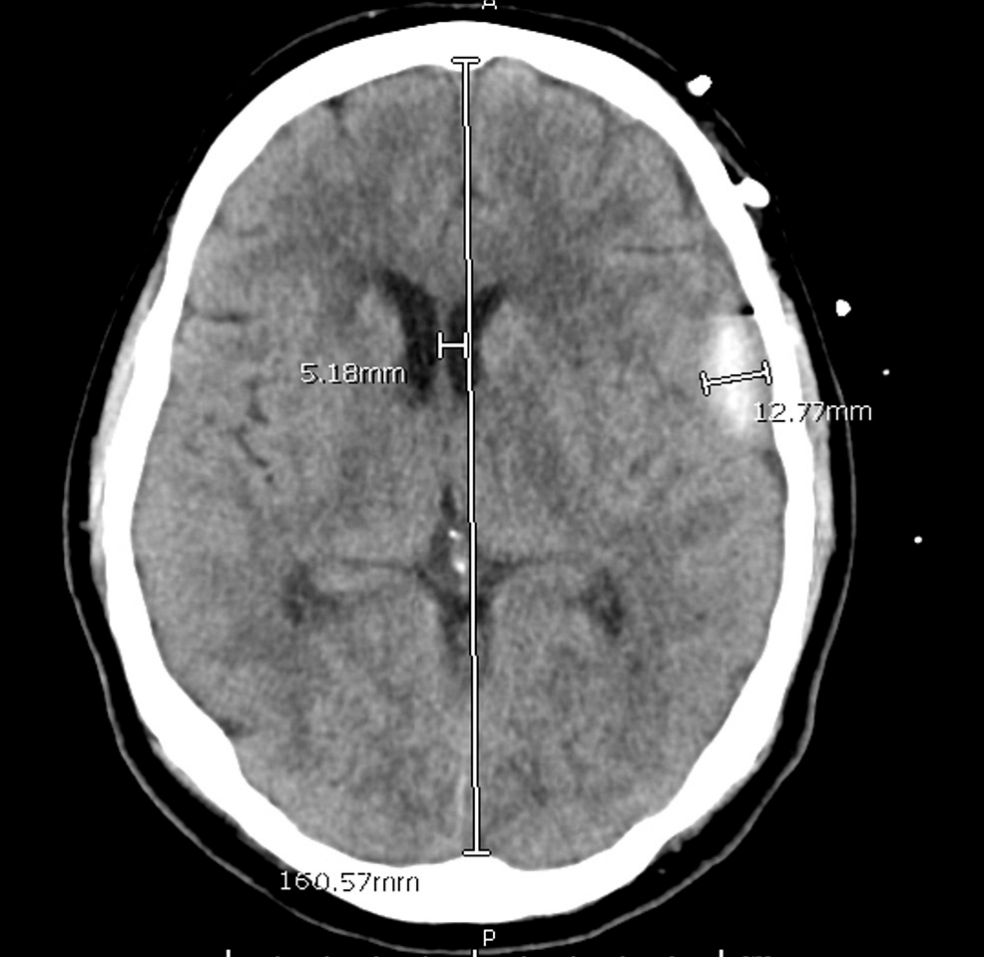
Post-operative Day One

**Figure 5:**
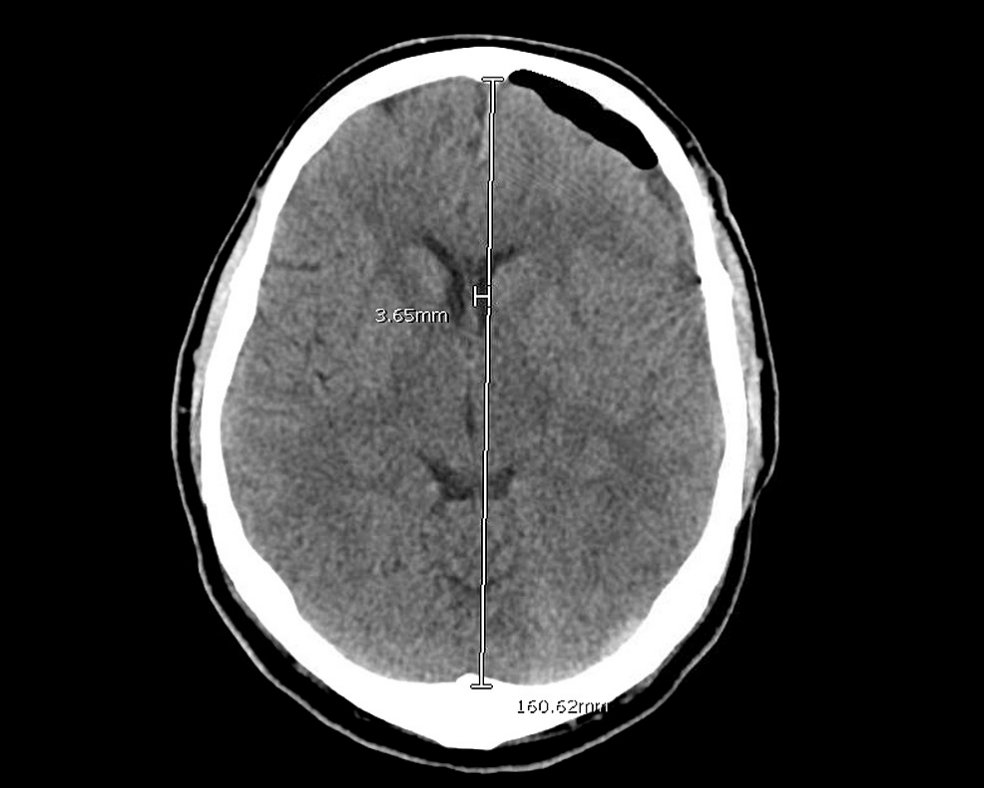
Post-operative Day Two Following IRRAflow Removal

Post-operatively the patient awoke from anesthesia without issue and was monitored closely in our neurocritical care unit. Daily CT scans were acquired and are displayed below (Figure 4, 5). His pre-operative speech deficits resolved following placement of the catheter. On postoperative day three the subdural had nearly fully resolved and he was discharged home from the intensive care unit. The patient has been seen in clinic twice since discharge with imaging studies. There have been no signs of recurrence at this time. 

## Discussion

In our opinion, cSDH can be viewed in two stages in terms of treatment: removal of compression and prevention of recurrent. Symptomatic cSDH can present with mental status changes, focal neurologic deficits, headaches, or seizures owing to mass effect and brain compression. In patients who present with these symptoms there is benefit in surgical evacuation in order to remove such compression [7]. As mentioned previously, recurrence is also a frequent issue in this pathology and MMA embolization has shown promise in resolving this issue. Given this treatment paradigm, our institution has adopted a practice in which patients undergo surgical intervention if symptomatic and this is followed by MMA embolization. 

Over the years there have been several techniques used to surgically evacuate cSDH. In essence they involve either a small craniotomy or burr hole placement over the hematoma. Data has shown that uncomplicated cSDH burr holes are associated with lower rate for recurrence and reduced morbidity. If cSDH are multiloculated, ossified, or have membranes, craniotomy can be considered [8,9]. Drains are typically placed in order to facilitate continued removal of the hematoma and to facilitate brain expansion. Subgaleal, subdural, and subperiosteal drains are possible options. Though subperiosteal drainage has been shown to have a lower complication rate than subdural drain placement, the recurrence rate is higher [10]. Duration of drainage ranges from 48-72 hours using standard drains [11]. 

MMA embolization is an emerging therapy in the treatment of recurrent cSDH. As mentioned previously, preliminary data has demonstrated low recurrence rates. However, much of the data is limited to patients who present with recurrence and were asymptomatic or minimally symptomatic [5]. The mechanism behind the effectiveness of MMA embolization relates directly to the underlying pathology. Usually there is some inciting cause such as tearing of bridging veins, coagulopathy, or intracranial hypotension. As the hematoma develops membranes begin to form. These membranes begin to form small capillaries that are easily disrupted leading to rebleeding and an ongoing inflammatory process [1]. MMA embolization is thought to disrupt the formation of these fragile membranes and thus decrease recurrence. This process is not instantaneous, limiting the use of MMA embolization to treat patients with symptoms severe enough to warrant admission to the hospital. 

Our treatment paradigm addresses both issues described above. Surgical evacuation allows the cause of symptoms to be safely removed while MMA embolization done prior to discharge helps to prevent readmission for recurrence. One issue remains the optimal surgical and drainage methods for patients. We prefer to use subdural drains attached to Jackson Pratt (JP) suction or drainage to gravity. One complication we have encountered with this process is failure of the drainage system leading to suboptimal drainage and brain re-expansion. As reported by Fargen et al., one factor that may influence this is the small caliber of many catheters [12]. Given the slow drainage that occurs to gravity or light suction, stagnation and subsequent clotting of blood is likely a contributing factor as well. 

Though new to the market, the use of self-irrigating catheters like the IRRAflow system shows promise in the treatment of cSDH [6]. This is no surprise given the benefits demonstrated when irrigation is used in addition to burr hole drainage of cSDH. One study by Ishibashi et al. demonstrated 2.9% recurrence rate when irrigation was used compared to 10.3% in burr hole drainage alone [13]. 

Our experience with this new treatment paradigm has been quite promising. Since the beginning of data collection we have performed MMA embolization and IRRAflow placement through a burr hole in four patients. Average length of stay has been 4.5 days from admission to disposition to home or rehabilitation. To date, there have been no recurrences or complications. One patient did have an extensive rehabilitation course and was lost to follow up as they died from cardiac comorbidities. Serial imaging was performed on this patient while in rehabilitation and did not demonstrate recurrence. 

## Conclusions

The placement of self-irrigating catheters using a burr hole in combination with MMA embolization is a safe and effective and minimally invasive treatment for cSDH. Preliminary data from our institution suggests an average length of stay of 4.5 days compared to six from standard surgical treatment. In addition, our recurrence rate is 0%, though this is significantly limited by a sample size of only four patients. Further research into the long-term safety and recurrence rates using this treatment paradigm need to be investigated with a case series. 
